# Surgical strategies and outcomes for myocardial bridges coexisting with other cardiac conditions

**DOI:** 10.1186/s40001-023-01478-9

**Published:** 2023-11-07

**Authors:** Mingkui Zhang, Xiruo Xu, Qingyu Wu, Hongyin Li, Zhonghua Xu, Hui Xue, Yongqiang Jin, Lixin Fan, Lina Li

**Affiliations:** Heart Center, First Hospital of Tsinghua University, Beijing, China

**Keywords:** Myocardial bridging, Myotomy, Coronary artery bypass grafting, Surgical treatment, Follow-up

## Abstract

**Background:**

Myocardial bridges are congenital coronary artery anomalies. There are still many controversies surrounding surgical treatment strategies for myocardial bridges combined with other heart disorders. The purpose of this study was to evaluate the surgical treatment strategies and outcomes in patients with these conditions.

**Methods:**

Between March 2004 and October 2021, our institution witnessed 77 patients diagnosed with myocardial bridging who underwent surgical intervention. According to the myocardial bridge and combined heart disorder, four groups were identified: 1. isolated LAD supra-arterial myotomy group, 2. LAD CABG and(or not) myotomy  group, 3. LAD supra-arterial myotomy and grafting of other branches group, and 4. LAD supra-arterial myotomy and other cardiac surgery group. The perioperative outcomes, symptoms, life quality, mortality, and major adverse cardiac events (MACEs) were analyzed.

**Results:**

There were no deaths during hospitalization and no rethoractomy for postoperative bleeding or major adverse cardiac events (MACEs). The follow-up period ranged from 2 months to 199.2 months (55.61 ± 10.21) months, the 10-year cumulative survival rates for the four groups of patients were 95.0%, 100%, 100% and 74.1%, and the 10-year freedom rates from the MACEs were 83.9%, 92.0%, 87.5% and 76.2%, respectively.

**Conclusions:**

Supra-arterial myotomy is preferred in patients with isolated myocardial bridge, and acceptable results can be achieved by choosing supra-arterial myotomy in combination with CABG or other cardiac surgery simultaneously for patients with myocardial bridges and other heart disorders.

## Introduction

Myocardial bridges (MBs) are congenital anomalies affecting the coronary arteries, characterized by a segment of a coronary artery, usually the left anterior descending branch (LAD), traversing the myocardium, thereby constituting a tunneled artery. The myocardium overlying the coronary artery forms the myocardial bridge. Historically, MBs were perceived as benign lesions; however, recent evidence indicates that patients with MBs may present with MB-related stable angina or acute coronary syndrome (ACS), ventricular arrhythmias, cardiogenic shock, and even sudden cardiac death [1, 2].

Notably, the compression of tunneled coronary arteries transpires not only during the cardiac systolic phase but also persists into the diastolic phase [2]. Furthermore, tunneled coronary segments exhibit a reduced predisposition to atherosclerosis, while accelerated atherosclerosis develops proximal to the bridged segment [3, 4]. Recent investigations have unveiled plaque formation in the proximal segment of the myocardial bridge in children with drug-refractory myocardial bridging, subsequent to intravascular ultrasound (IVSU) examination [5]. Consequently, MBs represent a risk factor for coronary atherosclerosis. In addition, MBs give rise to the branch steal phenomenon, leading to diminished septal blood flow [6]. MBs may coexist with congenital or acquired heart disease [7, 8], and demonstrate a higher prevalence in patients with hypertrophic cardiomyopathy [9].

For symptomatic patients, pharmacological management involving β-blockers or non-dihydropyridine calcium-channel blockers is the preferred treatment approach [10]. However, percutaneous coronary intervention (PCI) associated with risks, such as stent fracture, stent perforation, and in-stent restenosis [11, 12]. In patients exhibiting symptomatic bridging refractory to medical therapy, the surgical intervention options include supra-arterial myotomy or coronary artery bypass grafting (CABG). Previous investigations have demonstrated that supra-arterial myotomy can yield satisfactory outcomes in isolated LAD MBs [13, 14]. Nonetheless, the optimal surgical treatment strategy remains contentious for patients with MBs concomitant with other heart diseases or those presenting with deep and extensive MBs [13–16].

The objective of this study was to conduct a retrospective analysis of the surgical approaches, perioperative and prognoses of MBs in conjunction with other cardiac diseases, with the aim of offering clinical insights into the surgical treatment strategies for MBs combined with other cardiac diseases.

## Patients and methods

### Study population and design

Following approval from the Ethics Committee of the First Hospital of Tsinghua University (No.20210021), we searched our institutional database for patients diagnosed with MB [1]. Our study comprised a cohort of 77 myocardial bridge patients who underwent surgical intervention during the period from March 2004 to October 2021. All patients underwent coronary angiography for the assessment of coronary artery disease diagnosis and the evaluation of myocardial bridge compression severity. Among this cohort, 31 patients presented with isolated LAD–MB, 25 patients exhibited with LAD–MB accompanied by a proximal LAD stenosis, 10 patients manifested with LAD–MB along with other coronary branch stenoses, and 11 patients demonstrated with LAD–MB in conjunction with other heart disorders. Clinical data were acquired from the electronic medical record system, imaging data, surgical management system, and outpatient follow-up system.

**Fig. 1:**
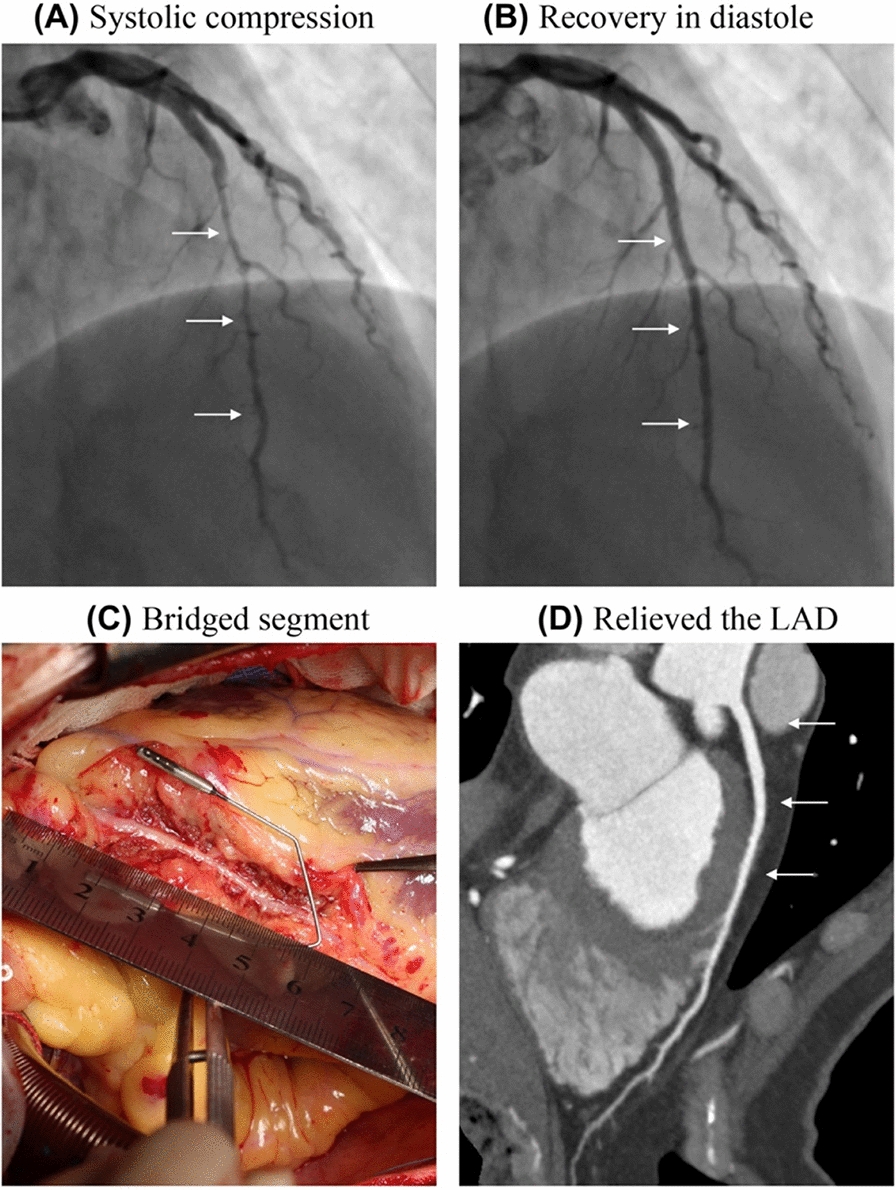
Coronary angiography before the surgery in a patient shows systolic compression (**A**, arrow), and recovery in diastole (**B**, arrow), bridged segment of left anterior descending artery unroofed (**C**). Coronary computed angiography shows completely relieved the left anterior descending artery myocardial bridge at 12-month follow-up (**D**, arrow)

**Fig. 2:**
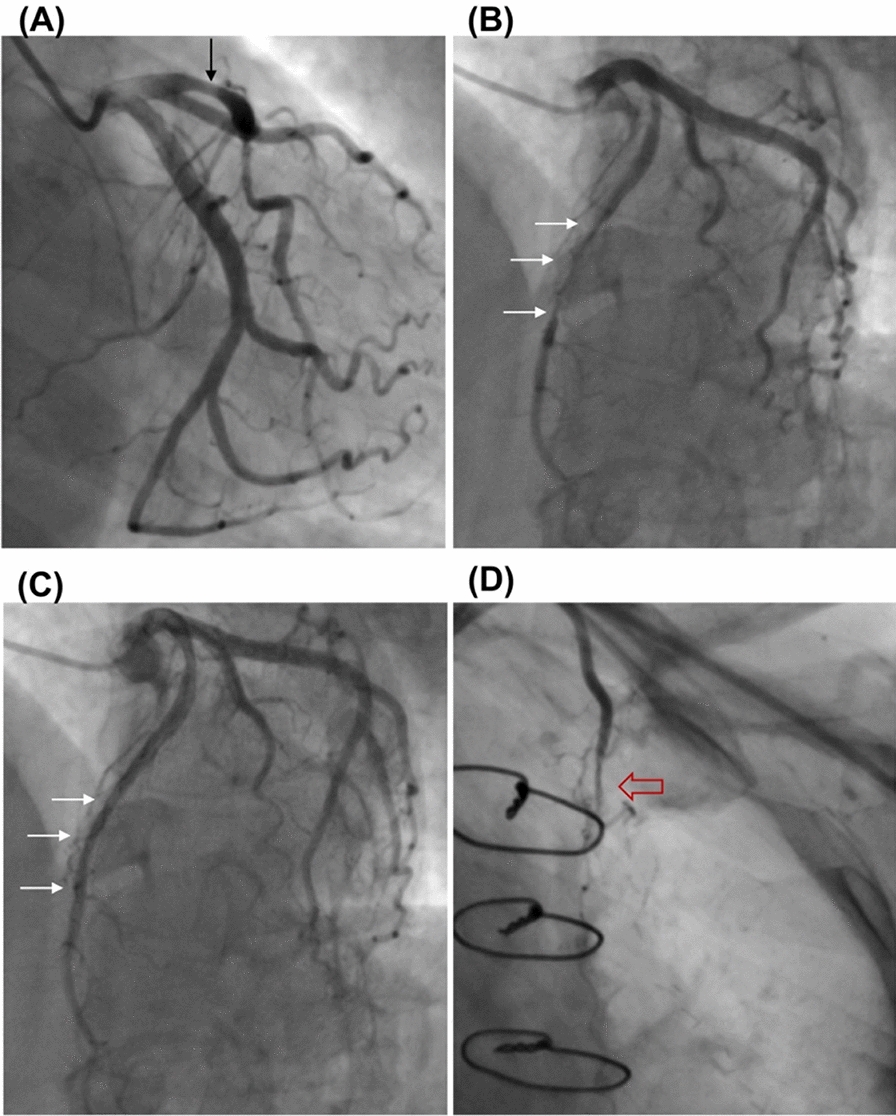
Coronary angiography shows LAD proximal coronary artery stenosis (**A**, black arrow), systolic compression (**B**, white arrow), recovery in diastole (**C**, white arrow) before the surgery, and a failed left internal mammary artery graft (**D**, red arrow)

Enrollment criteria were as follows: Group 1—isolated LAD supra-arterial myotomy: LAD–MB compression ≥ 70%, and angina symptoms resistant to medical therapy (Fig. 1A, B); Group 2—LAD CABG and(or not) myotomy group: individuals presenting with LAD–MB compression of ≥ 50%, in conjunction with LAD proximal coronary artery stenosis of ≥ 70%, may or may not have concomitant stenoses in other coronary arteries (Fig. 2A); Group 3—LAD supra-arterial myotomy and grafting of other branches: LAD–MB compression ≥ 70% and stenosis ≥ 70% in other coronary artery branches; and Group 4—LAD supra-arterial myotomy and other cardiac surgeries: LAD–MB compression ≥ 50% and coexisting heart disease necessitating surgical treatment.

### Surgical techniques

All procedures were executed by the three senior cardiac surgeons. For isolated supra-arterial myotomy, a median sternotomy or a small left anterolateral incision was employed, with or without extracorporeal circulation. The muscle overlying the anterior descending branch was incised using a small circular knife, fully relieving coronary artery compression (Fig. 1C). During the procedure, it was imperative to avoid injury to the anterior descending and diagonal branches, as well as to prevent right ventricular damage or rupture. Hemorrhage from muscle cut edge was managed with low-power electrocoagulation, while bleeding from small vessels was controlled using titanium clips or 6/0 polytetrafluoroethylene sutures.

CABG procedures were performed via a median sternotomy. The decision to perform the procedure with or without cardiopulmonary bypass (on-pump or off-pump) was contingent on the anatomical characteristics of the myocardial bridging. For cases of LAD arteriosclerotic stenosis in conjunction with myocardial bridges, the left internal mammary artery (LIMA) was employed as the graft vessel. For multiple coronary lesions, the saphenous vein was utilized as the graft vessel. In diffuse myocardial bridging scenarios, CABG was conducted subsequent to the myocardial bridging myotomy. For on-pump CABG, cardiopulmonary bypass (CPB) was established using right atrial and aortic cannulation, aortic cross-clamping, and the administration of cold-blood cardioplegic arrest. During off-pump CABG, the target coronary artery was stabilized with a tissue stabilizer (Octopus, Medtronic Corporation, Minneapolis, MN, USA).

In cases of LAD myocardial bridges accompanied by other heart diseases (heart valve disease, hypertrophic obstructive cardiomyopathy, etc.), myocardial bridge myotomy and cardiac surgery were performed concurrently.

### The seattle angina questionnaire

Seattle angina questionnaire (SAQ) is a rigorously validated self-administered instrument encompassing five functional status dimensions: physical limitation due to angina, angina stability, angina frequency, treatment satisfaction, and quality of life [17]. Utilizing the SAQ, clinicians can assess patients' quality of life and functional status preoperatively and during postoperative follow-up.

### Follow-up and study endpoints events

All patients underwent comprehensive follow-up, which was conducted via telephone, outpatient interview, and electronic medical records. The follow-up evaluations encompassed clinical symptoms, SAQ, pharmacotherapy, electrocardiography, echocardiography, coronary angiography, and coronary computed tomography (Fig. 1D). Major adverse cardiovascular events (MACEs) defined as the composite event of death, stroke, myocardial infarction, revascularization procedures and serious heart failure events [18].

### Statistical analysis

The data were processed using IBM SPSS 27.0 (IBM Corp., Armonk, NY, USA). The normality of the data distribution was assessed by the Kolmogorov–Smirnov test. Non-normally distributed were presented as median (interquartile range [IQR]). Normally distributed quantitative data were expressed as mean ± standard deviation $\left( {\overline{{\text{x}}} \, \pm \,{\text{SD}}} \right)$. Patients' pre- and post-operative SAQ scores were compared using a paired *t* test. Categorical data were expressed as frequencies or percentages, with differences deemed statistically significant at *P* < 0.05. The Kaplan–Meier curve was employed to delineate the all-cause mortality rate, and the incidence of MACEs.

## Results

### Patient demographic

The study included 77 patients, comprising 60 males and 17 females with a mean age of 55.2 ± 9.74 years. Comorbidities included hypertension in 21 (27.3%) patients, diabetes mellitus in 12 (15.6%) patients, hyperlipidemia in 23 (29.9%) patients, a history of myocardial infarction in 17 (22.1%) patients, and smoking in 33 (42.9%) patients. All patients underwent preoperative angiography and cardiac echocardiography, revealing a median left ventricular ejection fraction of 65(IQR 60–67). The patients’ baseline clinical characteristics are presented in Table 1.

**Table 1:** Baseline characteristics of all patients

Characteristics	Total (*n* = 77)	Isolate myotomy (n = 31)	LAD CABG and (or not) myotomy (*n* = 25)	Myotomy and other branch bypass grafting (*n* = 10)	Myotomy and other procedures (*n* = 11)
Age (years)	55.2 ± 9.74	54.55 ± 12.84	59(50.5–62)	54.5 ± 6.06	57.82 ± 9.28
Gender (female/male)	17/60	8/23	6/19	0/10	3/8
Symptoms (n)					
Chest pain (n)	66	31	25	10	0
Dyspnea (n)	27	10	7	4	6
Obesity (BMI > 30kg/m^2^)	5	1	2	1	1
Recent smoking (n)	33	12	12	4	5
Diabetes mellitus (n)	12	5	6	1	0
Hypertension (n)	21	11	2	5	3
Dyslipidemia (n)	23	10	8	5	0
Previous MI (n)	17	4	10	3	0
History of arrhythmia (n)	2	0	1	0	1
NYHA class III and IV (n)	3	2	0	0	1
CCS class III and IV (n)	23	15	3	5	0
Angiographic findings					
LAD MB (n)	73	31	21	10	11
Combined LAD stenosis (n)	21	0	21	0	0
Combined LAD obstruction(n)	4	0	4	0	0
Other branch stenosis (n)	29	0	20	9	0
Other branch obstruction (n)	4	0	3	1	0
Systolic compression degree of MB (%)	80(60–80)	80(80–90)	50(50–70)	75(60–80)	70(50–80)
LV EF (%)	65(60–67)	65(60–67)	60(60–66)	62.90 ± 6.01	60(60–69)
LVEDD (mm)	47.08 ± 5.15	45.29 ± 4.28	48.60 ± 4.86	46.70 ± 4.47	49.00 ± 4.20

Non-normally distributed were presented as median (interquartile range [IQR])

BMI: body mass index; NYHA; New York Heart Association; CCS: Canadian Cardiovascular Society; MI: myocardial infarction; LAD MB: left anterior descending myocardial bridge; LVEF: left ventricular ejection fractions; LVEDD: left ventricular end-diastolic dimension; CABG: coronary artery bypass grafting

### Surgical outcomes

There were no instances of surgical complications, such as coronary injury or ventricular rupture, nor recurrent angina during the perioperative period. CABG was performed in 35 patients, including 25 patients receiving LIMA–LAD bypass and 29 patients with saphenous vein grafts for other target vessels with multiple lesions. The outcomes for each patient group are detailed in Table 2.

**Table 2:** Procedure characteristics

Variables	Isolate myotomy (n = 31)	LAD CABG and(or not) myotomy (n = 25)	Myotomy and other branch bypass grafting (n = 10)	Myotomy and other procedures (n = 11)
Length of MB (mm)	40.32 ± 17.43	31.08 ± 15.33	54.00 ± 15.95	28.64 ± 11.20
Depth of MB (mm)	5(4–10)	4(3–5)	5.70 ± 2.21	4.64 ± 1.75
With CPB	22	18	10	11
CPB time (minutes)	71.50 ± 26.41	158.61 ± 56.21	79.00 ± 23.73	114.64 ± 50.48
ACC time (minutes)	43.86 ± 22.50	110(54.5–127.25)	52(40–60)	85.36 ± 43.19
Without CPB	9	7	0	0
Procedure				
Myotomy	31	17	10	11
LAD Bypass Grafting	0	25	0	0
D1 Bypass Grafting	0	9	5	0
LCX Bypass Grafting	0	10	3	0
RCA Bypass Grafting	0	10	2	0
Concomitant Procedures				
Mitral Repair	0	0	0	2
Mitral Valve replacement	0	0	0	5
Bi-valve replacement	0	0	0	2
Morrow Procedure	0	0	0	1
Cardiac myxoma	0	0	0	1

CPB: cardiopulmonary bypass; ACC: aortic cross-clamping; LAD: left anterior descending artery; LCX: left circumflex; RCA: right coronary artery

### Postoperative and follow-up outcomes

During their hospital stay, all patients remained free from adverse cardiac events, including acute infarction, heart failure, and ventricular arrhythmias. Over the course of follow-up period, two individuals in the LAD coronary artery bypass group were readmitted due to postoperative recurrence of angina pectoris myocardial ischemia symptoms. In one case, angina pectoris and additional symptoms of myocardial ischemia emerged 18 months after CABG (LIMA–LAD), but subsequently improved with pharmacological intervention. In another patient who had LAD–MB and proximal LAD stenosis on preoperative coronary angiography (Fig. 2A, B and C) and underwent LIMA–LAD grafting, angina symptoms recurred 12-month post-surgery, with coronary angiography revealing a failed LIMA graft (Fig. 2D). A patient in this group also underwent debridement for wound infection 3 months after the procedure. Hospitalization and follow-up results for the patients are detailed in Table 3.

**Table 3:** In-hospital and follow-up outcomes

Variables	Isolate myotomy (n = 31)	LAD CABG and(or not) myotomy (n = 25)	Myotomy and other branch bypass grafting (n = 10)	Myotomy and other procedures (n = 11)
In-hospital outcomes				
Mechanical ventilation time(h)	9(6.46–14.63)	7(5.5–12)	8.07 ± 2.89	13.53 ± 5.81
Reoperation for bleeding	0	0	0	0
Pleural fluid drainage at 24 h postoperatively(mL)	382.32 ± 159.50	487.40 ± 219.78	385(268–823.25)	338(274–408)
Total ICU stay (days)	1.74(0.85–1.9)	1.81(1.68–3.75)	1.78(0.98–1.93)	1.66 ± 0.67
New onset of acute MI	0	0	0	0
Atrial fibrillation	0	1	0	0
In-hospital deaths	0	0	0	0
Follow-up outcomes				
Duration of follow-up (months)	109.7(74.2,146.5)	85.05 ± 66.42	118.33 ± 54.38	111.92 ± 49.62
All-cause mortality	1	0	1	2
Cardiac death	0	0	0	0
Myocardial infarction	0	0	0	0
Repeat revascularization	0	1	0	0
Heart failure	0	0	0	0
Ventricular arrhythmia	0	0	0	0
Deep sternal wound Infection	0	1	0	0
CCS class III and IV	0	2	0	0

ICU: intensive care unit; CCS: Canadian Cardiovascular Society

**Fig. 3:**
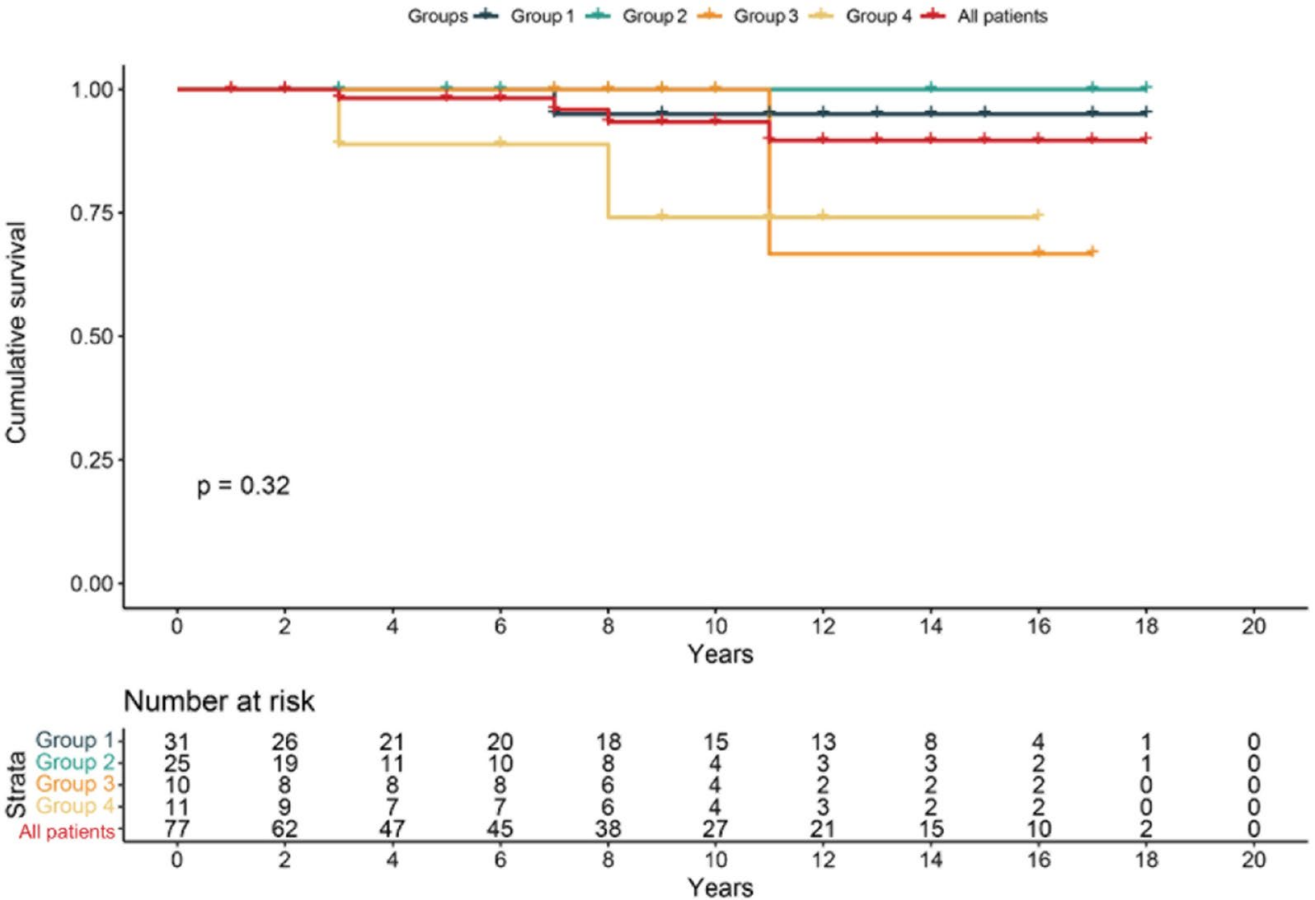
Kaplan–Meier survival curve of all postoperative patient during follow-up. Four groups were identified: 1. supra-arterial myotomy group, 2. coronary artery bypass grafting (CABG) group, 3. supra-arterial myotomy and other branches CABG group, and 4. LAD supra-arterial myotomy and other cardiac surgery group

**Fig. 4:**
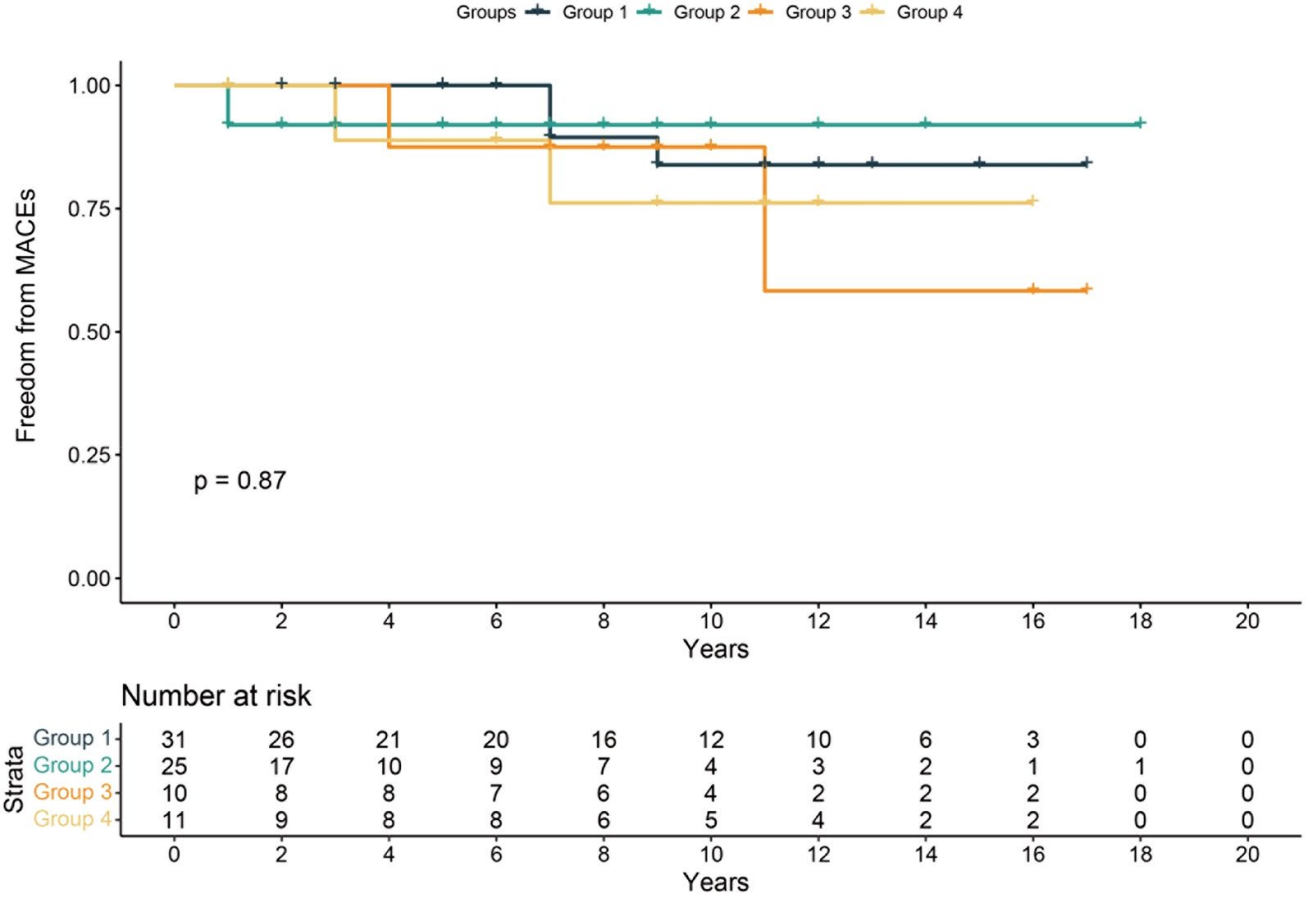
Freedom from MACEs curve for all the postoperative patients. The grouping in Fig. 4 replicates the configuration depicted in Fig. 3

Among the 77 cases, 59 were successfully followed up for an average duration of 55.61 ± 10.21 months. Notably, 18 cases were lost to follow-up (follow-up data were unavailable for 9 participants in Group 1, 5 in Group 2, 2 in Group 3, and 2 in Group 4.), potentially due to the considerable population mobility in China and its extensive territorial expanse. Moreover, meticulous statistical analysis has substantiated the absence of statistically significant variances in baseline characteristics, preoperative symptoms, and examination findings between the patients successfully followed up and those lost to follow-up. The 10-year cumulative survival rates for the four groups of patients were 95.0%, 100%, 100% and 74.1%, respectively (Fig. 3). In group 1, a patient died of cerebral hemorrhage 7 years after surgery. In group 3, a patient died of liver cancer 12 years after surgery. Similarly, in group 4, one patient died of cerebral infarction 3 years after surgery and another patient died of sudden death 8 years after surgery. The 10-year freedom rates from the MACEs for the four groups of patients were 83.9%, 92.0%, 87.5% and 76.2%, respectively (Fig. 4).

### SAQ results

In this study, we collected SAQ scores from the week leading up to the surgery until the final follow-up after the surgery. Post-surgical scores for physical limitation due to angina, angina stability, angina frequency, treatment satisfaction, and quality of life were significantly elevated in comparison with pre-surgical scores across all five dimensions assessed by the SAQ (*p* < 0.01, Table 4).

**Table 4:** Seattle angina questionnaire scores before and after surgery

	Mean	SD	SE Mean	95%CI	*T* value	*P* value
Isolate Myotomy						
Physical limitation due to angina	− 14.79	12.33	2.63	(− 20.26, − 9.32)	− 5.63	< .01
Anginal stability	− 56.81	24.62	5.25	(− 67.73, − 45.90)	− 10.85	< .01
Anginal frequency	− 40	26.55	5.66	(− 51.77, − 28.23)	− 7.07	< .01
Treatment satisfaction	− 37.68	− 16.01	3.41	(− 44.58, − 30.58)	− 11.04	< .01
Quality of life	− 31.06	12.39	2.64	(− 36.56, − 25.57)	− 11.76	< .01
LAD CABG and(or)Myotomy						
Physical limitation due to angina	− 17.66	8.17	1.78	(− 21.38, − 13.94)	− 9.91	< .01
Anginal stability	− 57.14	25.18	5.49	(− 68.6, − 45.68)	− 10.40	< .01
Anginal frequency	− 43.33	33.07	7.22	(− 58.38, − 28.28)	− 6.01	< .01
Treatment satisfaction	− 41.02	17.14	3.74	(− 48.82, − 33.22)	− 10.97	< .01
Quality of life	− 40.46	17.13	3.73	(− 48.25, − 32.67)	− 10.83	< .01
Myotomy and Other Branch Bypass grafting						
Physical limitation due to angina	− 23.50	13.13	4.96	(− 35.64, − 11.36)	− 4.74	< .01
Anginal stability	− 60.71	31.81	12.02	(− 90.13, − 31.29)	− 5.05	< .01
Anginal frequency	− 35.71	23.70	8.96	(− 57.63, − 13.79)	− 3.99	< .01
Treatment satisfaction	− 40.37	25.71	9.72	(− 64.15, − 16.59)	− 4.15	< .01
Quality of life	− 31.06	12.39	2.64	(− 36.56, − 25.57)	− 11.76	< .01

## Discussion

Our study substantiates that in isolated LAD myocardial bridges exhibiting ≥ 70% systolic compression, supra-arterial myotomy effectively alleviates vascular compression of the myocardial bridge, resulting in a significantly amelioration of the patient’s angina symptoms. For patients with coronary stenosis proximal to the myocardial bridge, CABG concomitant with supra-arterial myotomy or CABG alone can yield favorable outcomes, contingent on the anatomical features of the myocardial bridging. In patients presenting with concurrent heart diseases and myocardial bridges, executing supra-arterial myotomy alongside other cardiac surgeries can mitigate myocardial ischemia induced by myocardial bridge compression, thereby diminishing the risk of perioperative and postoperative myocardial ischemic adverse events.

In the context of isolated LAD myocardial bridges, our findings demonstrate that supra-arterial myotomy can effectively relieve compression exerted by myocardial bridges, mitigating myocardial ischemic symptoms. Postoperative coronary angiography or coronary CT revealed no residual myocardial bridge compression [14]. Given that myocardial bridging myotomy anatomically directly corrects the myocardial bridge compression, it is theoretically more appropriate for patients with myocardial bridges than CABG. Previous research has indicated a 2.6-fold increased risk of MACEs following CABG compared to myocardial bridging myotomy suggesting a superior long-term prognosis for the latter [19]. In a retrospective study, Ji et al. [16] observed that among 54 patients with myocardial bridges (31 cases myotomy vs. 23 cases CABG), the incidence of adverse cardiac events post-myocardial bridge myotomy was markedly lower than that of CABG, with a follow-up period of 26 months. In the current study, we encountered a case of LAD myocardial bridge (90% compression) with recurrent myocardial ischemic symptoms 2-year post-CABG (LIMA–LAD) at another institution. Coronary angiography detected occlusion of the bridging graft. In addition, occlusion of the bridging graft was detected on coronary angiography. We subsequently performed myocardial bridge myotomy, and the patient remained free of myocardial ischemia symptoms and adverse cardiac events at 10-year follow-up [20].

There is ongoing debate about optimal surgical approach for myocardial bridges myotomy particularly whether CPB should be utilized. Some studies have demonstrated satisfactory outcomes in selected patients, undergoing minimally invasive beating-heart MB myotomy [21, 22]. Conversely, other investigations have indicated that performing myocardial bridging myotomy under CPB may help prevent damage to the coronary arteries and right ventricle [19, 23]. In our cohort of patients undergoing isolated LAD supra-arterial myotomy, nine patients were treated off-pump and 22 were treated on-pump. Our findings support the use of on-pump surgery to fully release coronary bridges while providing superior protection to the coronary arteries and minimizing surgical complications, particularly for patients with deep and extensive MBs.

The surgical management of myocardial bridges combined with multiple coronary artery lesions has been infrequently reported. Evidence suggests that coronary arteries within MB segments are less susceptible to atherosclerosis, whereas those proximal to MBs are more predisposed to sclerotic stenosis [24]. High shear forces expose the intima of MB segments, leading to alterations in endothelial cell morphology and reduced expression of vasoactive proteins, such as nitric oxide synthase, endothelin-1, and angiotensin-converting enzyme. Consequently, the vessels within the MB are more vulnerable to spasm and thrombosis, while the coronary arteries proximal to the MB are more prone to plaque stenosis [25–27]. For patients exhibiting proximal stenosis of ≥ 70% in the LAD MB and ≥ 50% MB compression, we elected to perform CABG. However, in cases with long MB segments, small distal vessel caliber, and challenges in selecting anastomosis sites for the target vessel, concurrent MB myotomy can be considered [28, 29]. Moreover, evidence has demonstrated the existence of a branch steal phenomenon in MB segments, with MB myotomy shown to improve septal perfusion and reduce myocardial ischemia [6]. In our study, 25 patients with LAD–MBs and concomitant proximal stenosis were included, encompassing five patients with isolated LAD lesions and 20 with multiple coronary artery lesions. Seventeen patients underwent CABG combined with complete LAD–MB myotomy. One patient experienced recurrent angina postoperatively, and coronary angiography revealed a failed LIMA graft, potentially attributable to blood flow competition.

The co-occurrence of heart valve disease and myocardial bridges has not been documented in existing literature. This investigation analyzed 9 cases of heart valve disease concomitant with myocardial bridges, consisting of 7 instances of mitral valve lesions and 2 cases involving both aortic and mitral valve lesions. Heart valve surgery combined with myocardial bridge compression ≥ 50% was deemed an indication for myocardial bridging myotomy. No adverse events related to myocardial ischemia were observed in the follow-up periods. In addition, epidemiological research has revealed a significantly higher incidence of hypertrophic obstructive cardiomyopathy (HOCM) with myocardial bridges compared to the non-HOCM population [30]. Wang et al. [31] deduced that concurrent septal myotomy for HOCM and myocardial bridging myotomy is both safe and efficacious. In this study, one case of HOCM combined with LAD–MB (LAD compression 90%) was identified. Both septal myotomy and myocardial bridging myotomy were executed simultaneously, with no subsequent adverse cardiac events, such as myocardial ischemia. Consequently, these procedures are recommended for HOCM in conjunction with myocardial bridge compression ≥ 50%.

The SAQ can assess patient response to treatment, functional status, and impact on quality of life [17]. A significant correlation was found between SAQ and angina classification. Nevertheless, SAQ is only applicable for evaluating patients' functional capacity and quality of life, rendering it unsuitable as a disease severity criterion [32]. Maeda et al. employed SAQ to gauge children's postoperative quality of life after myocardial bridging myotomy, concluding that the procedure can alleviate symptoms unresponsive to medical therapy [33]. In the current study, postoperative SAQ scores demonstrated significant improvement compared to preoperative scores, signifying enhanced postoperative angina symptoms and quality of life.

This study's limitations stem from its single-center retrospective nature and small patient cohort, potentially introducing errors and constraints. First, only a minor fraction of patients underwent postoperative coronary angiography or coronary CT, impeding hemodynamic evaluation. Second, the absence of IVSU or fractional flow reserve (FFR) to assess the degree of systolic compression and hemodynamic changes in the coronary arteries is a drawback of this study. Third, surgical decisions were grounded in clinical experience rather than randomized control, and patients' postoperative complaints were subject to various factors. These factors could influence study outcomes. Consequently, further multi-center investigations and hemodynamic assessment are required to explore the surgical treatment strategy for myocardial bridges concomitant with other cardiac diseases.

In conclusion, this investigation establishes that supra-arterial myotomy is the recommended therapeutic approach for patients presenting with isolated LAD MBs. For patients exhibiting LAD MBs in conjunction with other cardiac pathologies, favorable outcomes can be attained by implementing a combined strategy of supra-arterial myotomy and concurrent cardiac surgical interventions.

## Data Availability

The data sets used and/or analyzed during the current study are available from the corresponding author on reasonable request.

## Abbreviations

CABG: Coronary artery bypass grafting
MACEs: Major adverse cardiac events
MBs: Myocardial bridges
LAD: Left anterior descending
ACS: Acute coronary syndrome
IVSU: Intravascular ultrasound
PCI: Percutaneous coronary intervention
LIMA: Left internal mammary artery
CPB: Cardiopulmonary bypass
SAQ: Seattle angina questionnaire
IQR: Interquartile range
HOCM: Hypertrophic obstructive cardiomyopathy
FFR: Fractional flow reserve
BMI: Body mass index
NYHA: New York Heart Association
MI: Myocardial infarction
LVEF: Left ventricular ejection fractions
LVEDD: Left ventricular end-diastolic dimension
CPB: Cardiopulmonary bypass
ACC: Aortic cross-clamping
LCX: Left circumflex
RCA: Right coronary artery
ICU: Intensive care unit
CCS: Canadian cardiovascular society
